# To Individualize the Management Care of High-Risk Infants With Oral Feeding Challenges: What Do We Know? What Can We Do?

**DOI:** 10.3389/fped.2020.00296

**Published:** 2020-06-09

**Authors:** Chantal Lau

**Affiliations:** Department of Pediatrics, Baylor College of Medicine, Houston, TX, United States

**Keywords:** evidence-based practice, feeding monitors, feeding tools, feeding guidelines, research-to-practice translation, feeding efficiency and safety, prematurity and low birth weight

## Abstract

The increase in preterm infants' survival over the last 30 years has shed light over their inability to feed by mouth safely and efficiently. With adverse events such as increased risks for oxygen desaturation, bradycardia, penetration/aspiration, infants' hospitalization in neonatal intensive care units (NICUs) are understandably prolonged. Unfortunately, this leads to delayed mother-infant reunion, maternal stress, breastfeeding obstacles, and increased medical costs. Such impediments have stimulated clinicians and researchers to better understand the underlying causes and develop evidence-based solutions to assist these infants. However, it is notable that the research-to-practice translation of this knowledge has been limited as there are still no validated guidelines or protocols as how to best diagnose and care for these infants. This report revisits the immature physiologic functions at the root of these infants' oral feeding difficulties, the current practices, and the recent availability of evidence-based efficacious tools and interventions. Taking advantage of the latter, it presents a renewed perspective of how management strategies can be tailored to the specific needs of individual patients.

## Introduction

Oral feeding difficulties in children is a *subtle* condition that is not a well-recognized public concern. It has been reported that 20–50% of healthy developing children encounter such complications (1, 2). This incidence can rise to 80% for children with developmental disabilities and complex medical conditions, such as prematurity, cerebral palsy (https://.asha.org/PRPSpecificTopic.aspx?folderid=8589934965&section=Incidence_and_Prevalence). In a recent study of moderate preterm infants [29^0/7^-33^6/7^ weeks gestational age (GA)] from 18 sites within the NICHD Neonatal Research Network, “inadequate oral feeding” was the most prevalent barrier to hospital discharge. Of the 56% (3,376/6,017) of infants who remained hospitalized until 36 weeks postmenstrual age (PMA), 37% (1,262 infants) were clinically stable “feeders and growers” whose only delayed discharge was due to inadequate oral feeding performance (3).

In neonatal intensive care units (NICUs), as the survival of infants born prematurely increased, it has come to light that for many of them, attainment of independent oral feeding is a struggle leading to prolonged hospitalization, while increasing maternal stress and medical cost (3–5). Such condition is often followed by re-hospitalization or visits to feeding disorder clinics as our current knowledge-based practice for the care of these infants has not kept up with their increased survival (5–8). This is reflected by the lack of validated and structured oral feeding guidelines at how to best manage these issues and the large variations in practices between hospitals (3, 9–11). As attainment of independent oral feeding is one of the three criteria infants need to meet prior to home discharge (5), the faster they can wean from tube feeding safely and efficiently, the sooner they can be reunited with their mothers. Such accomplishment is based on their ability to complete all their feedings (breast or bottle) with no adverse events, e.g., oxygen desaturation, bradycardia, within an allotted period of time, e.g., 20–30 min to avoid excessive energy expenditure, while demonstrating appropriate weight gain, e.g., ~15 g/kg/day.

The purpose of this report is to present a review of the current practices and the efficacious research tools/interventions developed over the last two decades shown to shorten time to safe and efficient attainment of infant independent oral feeding. In combining this research knowledge with current clinical practices, a novel feeding management plan/guidelines catered specifically to the care of *individual* infants in NICUs is presented.

## What Do We Know?

### Immature Physiologic Functions Are at the Root of Preterm Infants' Oral Feeding Difficulties

Generally, in clinical practice, the evaluation of a particular patient's condition(s) begins by conducting a *differential diagnosis* based on the systematic review of possible causes before his/her management plan is developed. However, it was not until the survival of preterm infants in NICUs increased that healthcare providers' concern over infant oral feeding difficulties came to light. As such there was a limited understanding of the neuro-physiologic and -motor components implicated in their ability to feed by mouth. This led to an increased interest in understanding the maturation of their oral feeding skills and in the development of tools and therapies to assist these young patients more readily wean from tube feeding. It is following the development of the oro-motor kinetic monitoring (OMK) technology that our laboratory began to understand the complex involvement of the different neuro-motor and -physiologic functions required for these infants to feed safely and efficiently (12–16).

**Figure 1:**
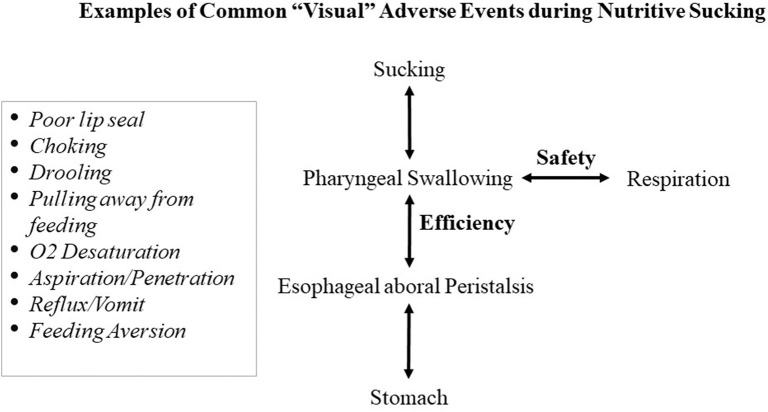
Commonality of “visual” adverse responses caused by untimely events at any level of the Swallow Process.

At present, for infants with oral feeding challenges, the focus has been primarily on infants' ability to suck, swallow, and breathe. However, amiss in this rationale is the role of the esophageal function (17, 18). Indeed, the transport of a bolus from the mouth to the stomach involves two elements that independently ensure efficiency and safety. Efficiency relates to the proper coordination of the phases of the Swallow Process; namely, the formation of the bolus during the oral phase, its swift transport through the pharynx during the pharyngeal phase, and through the esophagus during the esophageal phase (19). Safety in swallowing, for infants as well as adults, relates to the proper timing of respiratory inhalation/exhalation during the pharyngeal phase of swallowing to prevent O_2_ desaturation and/or liquid aspiration/penetration into the lungs during inhalation (20, 21). The difficulty in diagnosing the origin(s) of oral feeding difficulties results from the fact that uncoordinated or improper execution at any phase of the Swallow Process can lead to the same *visual* adverse responses shown in Figure 1, e.g., drooling, poor lip seal, oxygen desaturation, pulling away, and feeding aversion. Consequently, based on the commonality of behaviors demonstrated by infants during oral feeding and the lack of appropriate tools to properly monitor the functionality of each phase of the Swallow Process and respiration, it is difficult for caregivers, as observers, to identify the cause(s) responsible for the onset of such events. It is for this reason that the current knowledge-based management plans devised by feeding therapists for bottle- and breast-feeders, i.e., occupational therapists, speech language pathologists, lactation consultants, raise concerns among their multi-disciplinary team members whose viewpoints understandably are influenced by their respective professional training and expertise. Additionally, consensus for best practice is often challenged by the use of qualitative/descriptive over quantitative/objective approaches. This is particularly germane when any resulting benefit may simply be due to infants' normal maturation (22). Other significant factors also impact preterm infants' oral feeding performance. Their abrupt transition from an *in-utero* to *ex-utero* environment forces them to adapt to conditions that they are not developmentally prepared for, e.g., bright lights, loud sounds, varying temperatures, unsupported forced postures (10). As basic physiologic/behavioral/organizational functions, e.g., sleep/awake, calm/agitated, mature at different times, studies have shown that exposure to such variety of NICU stressors has been correlated with changes in infant brain structures and functions (23, 24). In brief, the multitude of maturational and environmental factors that lead preterm infants to exhibit similar adverse events during oral feeding makes it i**s** difficult to identify the culprit(s) at the origin of their oral feeding difficulties.

### Current Knowledge-Based Practice Guidelines, Tools, and Interventions

When advancement of infant oral feeding in NICUs is hampered, feeding therapists are commonly consulted (19, 25, 26). Due to the lack of available devices to monitor infants' sucking skills, non-nutritive assessment follows standardized plan based on visual and sensory feedback whether infants are bottle- or breast-feeding. Using a gloved finger, therapists assess the anatomical development of infants' oral structures, e.g., hard/soft palate, gum line, and lingual mobility, e.g., lateralization, cupping, stripping. As the infant is sucking on the therapist's gloved finger, functional development is assessed by visual and tactile feedback, i.e., watching the movement and coordination of cheeks, jaws, and lip seal around the finger, the relative rhythmicity of pressure forces applied on the finger, and respiratory bursts/pauses. Evaluation of nutritive sucking skills conducted during bottle feeding is based on similar visual assessment of cheeks, jaws, lips, and the relative coordination of suck, swallow, and respiration while watching for any adverse events, e.g., choking, O_2_ desaturation, pushing back (12). As infant organization/states or external factors such as NICU surroundings, may interfere with performance, a management plan that encompasses the above concerns is proposed that may include varied perioral stimulations, e.g., cheek and chin support, pacing, non-nutritive oral motor stimulation (NNOMT), adjusting feeding positions, e.g., sidelying vs. semi-reclined, dimming overhead lights, etc. (10, 27, 28). In recent years, responsive/cue-based/“infant driven feeding” approaches have grown in popularity. However, in the absence of data providing strong evidence of study design stringencies, a recent Cochrane Intervention Review recommended that “a large RCT [randomized controlled trial] would be needed to confirm “their benefits and determine if such approach may affect other important [preterm infants'] outcomes” (29, 30). When flow rate during bottle feeding is deemed too slow or too fast, it is normally addressed by changing nipples claimed to be slower/faster as per manufacturers (31–34). Insofar as caregivers' decisions are subjective and use trial and error approaches, at times, there lacks a general consensus among the multi-disciplinary members of the NICU team regarding best treatment (27, 28, 35, 36).

The health benefits gained by mothers and infants through breastfeeding are no longer disputed (37–40). In addition to the nutritional advantage mother's milk offers over artificial formulae, breastfeeding is the optimal nurturance infants can receive from their mother through their close physical contact. Indeed, a mother's balanced nutritional and maternal care will benefit her child's not only nutritionally, but also non-nutritionally in the provision of appropriate stimulation of their infants' maturing neuro-physiologic/-motor/-behavioral functions (41–46). Breastfeeding challenges for NICU infants has similarly increased the demand for lactation consultants. Evaluation for breastfeeding difficulties assesses maternal factors e.g., nipple shape, degree of elasticity/protractility as they may interfere with infant's performance and ability to latch-on and remain latched-on during a feeding (6). As breastfeeding requires the involvement of both mother and infant, when infant breastfeeding difficulties arise, any evaluation requires not only assessment of infant oral feeding skills, but just as importantly the lactation performance of their mother. It remains unclear whether a mother's mammary development and function is affected by her shortened gestation. Lactation may be impaired not only by milk supply and milk release during breastfeeding, but also by mother's motivation to breastfeed, her overall well-being, and stress (41, 47). It is well-acknowledged that stress can interfere with the neuro-endocrine regulation of lactation both at the level of milk synthesis and milk release/ejection (6, 41, 46). Although a number of breastfeeding assessment scales have been developed based on a variety of infant behavioral criteria, they are not yet well-recognized (48–53). This situation is similar to that of feeding therapists' evaluation of infant's bottle-feeding difficulties as lactation consultants' feedback lack objective evidence-based studies supporting their proposed treatment(s). Nevertheless, the broad variety of current knowledge-based bottle and breastfeeding approaches proposed by feeding therapists underscores “the importance of strategies for stimulation of [infant] sensory-motor-oral system to decrease the period of transition to full oral feeding system” (35).

### Novel Research Tools/Interventions

A small number of devices have been developed to monitor infant non-nutritive and nutritive sucking. As described on their respective website, the NTrainer system (innarahealth.com) specifically monitors infants' non-nutritive suck and has developed non-nutritive assessments and therapies that “have been proven to reduce time to full oral feeding […] and length of stay […] in the NICU1.” The NFANT® Sensor (nfant.com) monitors infant lingual movements during feeding and assist clinicians to “quickly determine optimal feeding parameters through objective metrics” using their Nfant Feeding Solution and Analytics. The Medoff-Cooper Nutritive Sucking Apparatus (M-CNSA) is a research tool that monitors sucking pressure (54).

Due to the commonality of the “visual” adverse symptoms that arise from dysfunctionality(ies) at the various levels of the Swallow Process (Figure 1), our approach attempted to examine the proper functionality at these levels. As our intention was first to specifically understand the development of infant nutritive sucking, our studies were conducted during bottle- rather than breast-feeding as the impact of any maternal inputs through their behavior and milk availability would obligatorily add non-controllable external variables that would not be indicative of their infants' *true* competence. As infants comorbidities, e.g., bronchopulmonary dysplasia, post necrotizing enterocolitis, could interfere with infants oral feeding performance, our subjects were recruited from *clinically stable* very low birth weight (VLBW) preterm infants, i.e., “feeders and growers,” whose discharge from NICUs were only delayed due to their inability to adequately feed by mouth (3, 13). Following the development of the oro-motor kinetic monitoring (OMK) technology, we gained a substantial understanding of the maturation of VLBW infants' nutritive and non-nutritive sucking and how appropriate synchronization of suck-swallow-breathe was necessary to ensure safe and efficacious oral feeding. With the understanding gained from the OMK technology, the development of additional tools and interventions naturally followed.

#### The Oro-Motor Kinetic Monitoring Device (OMK) Technology

##### The OMK Nutritive Assessment Strategy (OMK-NS)

To follow the natural development of infant nutritive sucking, the OMK-NS was developed using miniature sensors appropriately placed on the bottle nipple. As such, it can *directly* monitor the shape/form and strength (mmHg) of the two components of sucking, i.e., Suction and Expression (55). Suction corresponds to the negative intra-oral pressure generated with the closing of the nasal passages by the soft palate and lowering of the jaw that draws milk into the mouth, similar to drinking from a straw. Expression, in turn, corresponds to the compression and stripping of the nipple by the tongue against the hard palate to eject milk into the mouth. With this device, we described five stages of nutritive sucking (NS) maturation based on the presence/absence of suction, expression, their respective rhythmicity, and synchronization with one another (16). We have also shown that this technology can be readily adapted to the breast nipple during breastfeeding.

**Figure 2:**
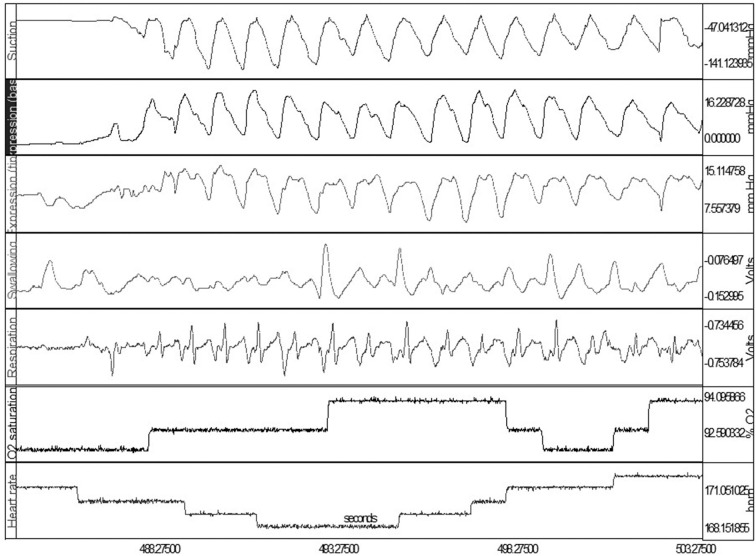
Simultaneous OMK-NS monitoring of nutritive sucking, i.e., suction, expression (base and tip), swallow, respiration, O_2_ saturation, heart rate. Note: Expression monitored at base and tip of tongue for assessment of lingual mobility, e.g., compression, stripping.

To simultaneously monitor the sucking-swallowing-breathing events during bottle feeding, two drums placed over the hyoid and diaphragm allowed for the capture the pharyngeal swallow reflex and respiratory effort (56), respectively. This provided evidence for the importance of the proper *temporal* coordination of sucking, swallowing, and respiratory functions (14, 16, 57, 58). If connected to infants' vital signs monitors, Figure 2 shows how one can monitor at the same time infants' clinical status.

The clinical importance of pediatric esophageal dysphagia is well-recognized by pediatric gastroenterologists (59). As the gastrointestinal (GI) system of the pig is the closest to that of the human (60), we conducted a study on preterm piglets using a 4-port “multi-channel” esophageal catheter provided by our co-author (Omari T) and obtained evidence supporting the importance of the proper maturation of esophageal peristalsis as another essential component for the safe and swift transport of a bolus from the oral cavity to the stomach (17). Preterm piglets demonstrated similar susceptibility to necrotizing enterocolitis and oral feeding issues as their human counterparts, e.g., milk leakage, regurgitation, limited endurance, inability to complete a feeding. When compared to healthy term piglets, the occurrence of the mature pattern of aboral propagating peristaltic waves for bolus transport, i.e., from upper esophageal sphincter to stomach, was significantly less frequent and of slower velocity (17). The development of monitoring devices has been problematic due to infants' fragility and small sizes. However, devices for the assessment of esophageal function, using high resolution manometry with and without impedance (HRIM/HRM) are becoming adaptable to the pediatric population (61–65).

##### The OMK Non-nutritive Assessment Strategy (OMK-NNS)

The OMK technology can be adapted for non-nutritive sucking assessment on a pacifier or a disposable glove as used by feeding therapists for their consults, providing objective quantitative measures similar to those obtained during nutritive sucking (OMK-NS) e.g., pressure force (mmHg) of suction and expression, NNS frequency, shape/form of these two components, duration of pauses and sucking bursts that cannot be identified otherwise (10, 12, 13). It is advanced that if used for the preliminary clinical evaluation of infant oral feeding skills, therapists' recommendations based on objective outcome measures would be more readily accepted by their NICU team members than currently viewed. Of value, the OMK-glove may be a valuable tool for the objective training of new feeding therapists as they can learn to interpret the variations of their sensory feedback from the recordings obtained. With the OMK technology, we confirmed that nutritive sucking occurs at 1 cps vs. that of non-nutritive sucking at 2 cps (66). This is a useful means to help determine if milk release is occurring when infants are nutritively vs. non-utritively sucking on the breast. When thickeners are added to formula/breastmilk during bottle feeding to decrease reflux, they may occlude the nipple hole preventing milk outflow. This can be readily recognized if infants begin sucking non-nutritively at 2 cps. Of interest, we have observed that infants' non-nutritive sucking is already mature by the time oral feeding is initiated. This implies that the oro-motor competence to generate suction and expression is already attained (12). For this reason, the observation of “rhythmic” sucking on a pacifier/finger is incorrectly used as an index of readiness to feed.

#### The Oral Feeding Skills Scale (OFS)

**Figure 3:**
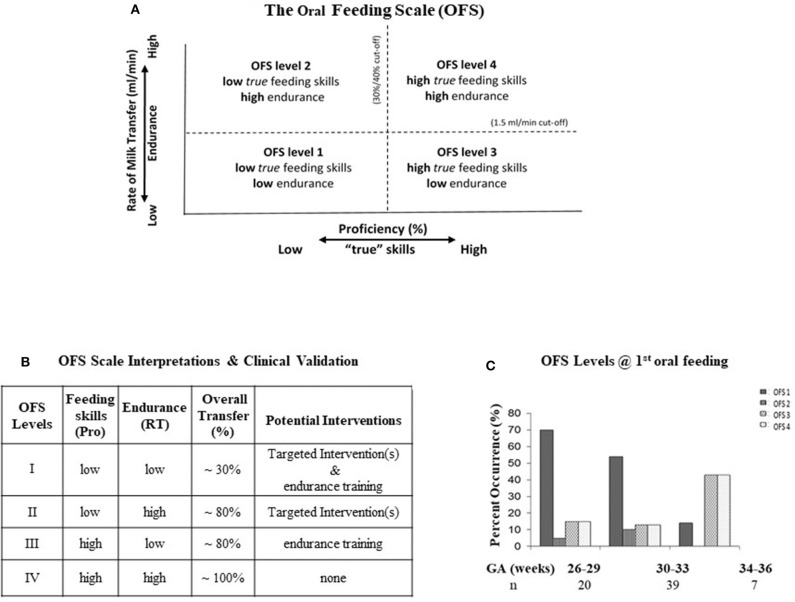
**(A)** The four OFS levels delineated by: Proficiency on the *X*-axis at 30 and 40% for VLBW and late preterm, respectively; Endurance on the *Y*-axis at 1.5 ml/min. **(B)** Interpretations and Clinical Validation of the OFS Levels, Feeding Skills (Pro: proficiency), Endurance (RT: rate of milk transfer), Overall transfer (% volume taken/volume to be taken), Potential targeted interventions for the respective “low” levels at each OFS levels (see Figure 5). **(C)** Percent occurrence of OFS levels (I–IV) in three groups of infants born at 26–29, 30–33, and 34–36 weeks GA at their first oral feeding (~35 weeks PMA); *n*: number of subjects/group. Note: broad variations of OFS levels at each GA.

The Oral Feeding Skills scale (OFS) is a 4-level *clinical* scale developed as a simple objective indicator of infants' feeding ability. It has the unique advantage that it does not require any special device, only the recording of the percent volume taken over volume prescribed during the first 5 min of a feeding (“proficiency”) and the rate of milk transfer (ml/min) over an entire feeding (“endurance”). The OFS assessment presumes that prior to all oral feedings, infants are left undisturbed 30 min prior in order to ensure minimal fatigue before they are fed. As shown in Figure 3A, proficiency on the *X*-axis is used as an estimate of infant's “true” feeding skills *as fatigue is minimal*. Rate of milk transfer on the *Y*-axis is used as an index of endurance or how fatigue impacts an infant's overall feeding performance (67). Four levels of OFS skills are delineated by cut-offs of proficiency set at 30% and “endurance” at 1.5 ml/min for VLBW infants (67), and 40% and 1.5 ml/min for late preterm, respectively (68). These four OFS levels distinguish proficiency (*true* feeding skills) and endurance levels as “high” or “low.” Figure 3B presents the levels of these two measures at each OFS levels, I–IV, and their correlation with infant overall feeding performance or overall transfer, i.e., percent volume taken/volume prescribed at a feeding. A significant correlation was observed between each OFS levels and % overall transfer (67). It is of interest to note that OFS levels II and III show that proficiency and endurance, on their own, have equal impact on overall transfer [~80%; Figure 3B]. Potential interventions comprising “targeted interventions” and “endurance training” to assist infants when tested at these individual OFS levels are based on their respective proficiency and endurance (Figure 3B). Figure 3C confirms that preterm infants can demonstrate the whole range of OFS levels I–IV when monitored at their first oral feeding (67). This would explain why, as caregivers, we are familiar with the disparity that infants of same GA and PMA may show broad ranges of oral feeding aptitudes.

The OFS scale offers several advantages: (1) It can be used at any feeding with no special device and without interfering with infant's task because taking a reading of the volume taken at ~5 min into a feeding coincides with an initial recommended “burping” pause. (2) As it is correlated positively with infants' % overall transfer during a feeding (Figure 3B), it becomes an objective index of the correlation between infants' oral feeding skills and performance over time. (3) The observation that infants of similar GA monitored at similar PMA demonstrate all four OFS levels confirms that their oral feeding skills mature at different rates (Figure 3C). As such, care should be taken not to interpret that infants falling below “expectation” are delayed. (4) With the ability to monitor the maturational progress of an *individual* infant's proficiency and endurance, the OFS offers the opportunity to differentiate the impact that the maturation of “true” skills (proficiency) and fatigue/endurance can have on infant overall performance (10). Consequently, infants' OFS levels are better indicators of oral feeding competence than the use of PMA or aptitude to suck on a pacifier. (5) The efficacy of an intervention on a particular infant can be verified as it would be reflected by improved OFS level along with corresponding oral feeding performance (10). Providers need to remember that if a particular intervention is not beneficial for one infant, it can be for another as the cause of their respective dysphagia may lie at different levels of the Swallow Process. (6) Finally, when used in patient rounds, the 4-level OFS scale offers team members an objective/quantitative feedback of a patient's performance over time that the current subjective/descriptive approaches cannot, e.g., “baby fed well,” “poorly,” “better/worse than the day before.” Due to its objective feedback, the OFS scale has been adopted into patient's medical records in some hospitals as well as in research.

#### The Infant Self-Pacing (ISP) Feeding Bottle

**Figure 4:**
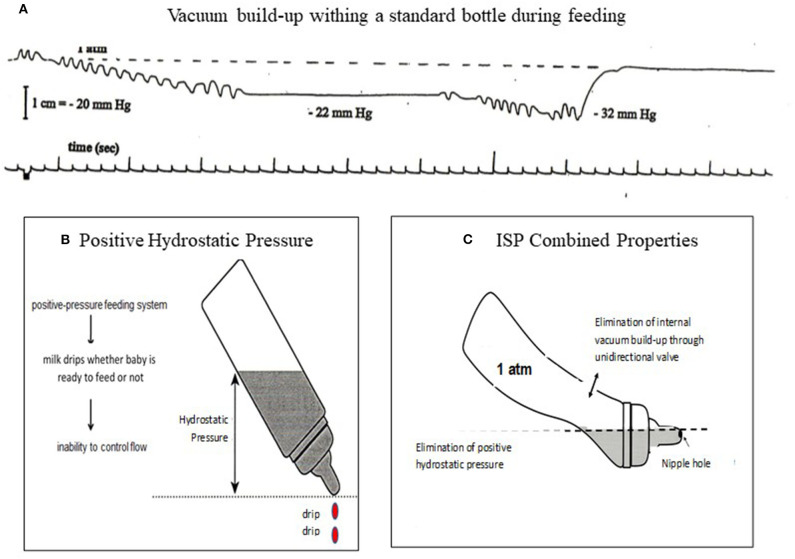
**(A)** Internal vacuum build-up from 1 atm (0 mmHg) to −22 mmHg after first sucking burst maintained as infant did not release the nipple, decreasing to −32 mmHg after a second sucking burst. Infant would need to generate a sucking force >32 mmHg in order to obtain milk. **(B)** a positive hydrostatic pressure over a nipple hole leads to a natural outflow (drip) whether infant is sucking or not. **(C)** ISP principles: Elimination of internal vacuum and hydrostatic pressure over nipple hole gives infants control over their own feeding.

The principles of the ISP bottle eliminate two properties of fluid physics that occur within a rigid standard bottle as it empties when an infant is feeding. As a standard bottle empties during a feeding, a natural increase in internal vacuum build-up occurs hindering fluid outflow. This leads infants to suck harder to first overcome the internal vacuum before obtaining milk, likely increasing unnecessary energy expenditure (Figure 4A). The positive hydrostatic pressure exerted by milk over the nipple hole when a bottle is tilted naturally leads to a disruptive “milk drip” whether the baby is sucking or not (69) (Figure 4B). With the elimination of the natural internal negative pressure build-up after each suck and “milk drip” (Figure 4C), the *control of the feeding is given to the infant* and not the caregiver because the infant does not need to suck harder (preserving energy) to overcome the increasing internal negative pressure and can pause to rest/catch-up breathing whenever needed without milk dripping in his/her mouth. Although the volume of a milk “drip” may not appear of significance, it should be noted that the average bolus size of VLBW infants average 0.14 ± 0.06 ml when they transition from tube to independent oral feeding. This is significantly less than the 0.22 ± 0.07 ml monitored in full term infants during their first 2 weeks of life (14).

In general, bottle feeding is controlled by caregivers who, through visual and auditory sensory feedback, determine whether a feeding ought to be maintained or stopped. Unless obvious adverse events occur, e.g., turning blue, pushing away, triggered hospital alarms, the feeding is continued. Caregivers may take different approaches in “assisting” their infant's feeding such as increasing/decreasing milk outflow by using fast or slow flow nipples, offering softer/harder nipples, providing “encouragement” to complete a feeding, or stopping the feeding because infant appears satiated. Under such circumstances, it is unclear the bases upon which such rationales arise because without appropriate devices, we know that immature neuro-motor/-physiologic events cannot be readily detected, e.g., dys-coordination of suck-swallow-respiration-esophageal motility, silent penetration/aspiration, non-overt gastro-esophageal reflux.

To our knowledge, the ISP is the only feeding bottle that gives *control of the feeding* to the babies. Whenever needed, e.g., rest, catch-up breathing, it allows them to stop with the bottle in the mouth without being overwhelmed as milk drip is also eliminated. This assumes no caregivers' inputs during feedings. With the ISP, we observed that a greater percentile of infants completed their feeding with no adverse events. This was achieved at a faster rate while using a more mature OFS level than control counterparts feeding from a standard bottle (69, 70). Therefore, we speculate that infants, *with normally developing central nervous system*, are able to *reflexively* regulate their nutritive sucking *in the absence of external controlling factors*. This assumption is based on anatomical evidence that the respiratory, sucking, and swallowing centers are anatomically in close proximity from each other in the brainstem, with separate pools of motor neurons implicated in their respective sequential rhythmic movements and regulated by central pattern generators (CPGs) (71, 72). The existence of an intrinsic “tau (τ)” guide acting as a common processor that links timing events of different motor movements has been proposed (73–75). A similar theory of cross-system interactions between suck, swallow, and respiration has been advanced to explain the ability of these functions to rapidly re-adjust to variations occurring at any one of these levels during oral feeding (76). The essential integrity of sensory afferents signaling changes in physiologic and environmental functions has also been proposed for the proper regulatory feedback of these individual functions (77).

From such understanding, the following tools along with evidence-based tested interventions we developed are described below.

#### Interventions That Enhance Infants' Oral Feeding Aptitude

Infant oral feeding *performance* does not solely relate to the proper maturation of infants' oral feeding skills. Their clinical status, behavioral states, infant's organization, and environmental conditions at feeding time are well-known contributors to a successful feeding (6). Oral feeding is optimized when infants are in drowsy/alert inactive, quiet awake, and/or alert state as defined by the Newborn Individualized Developmental Care and Assessment Program (NIDCAP) (78–80). As mentioned earlier, environmental conditions such as bright light, loud surroundings, fluctuating temperatures, infant unsupported posture are disruptive (10, 81). Consequently, the development of any intervention needs to encompass not only uni-modal approaches targeting physiologic functions, e.g., sucking, swallowing, respiration, esophageal function, but also multi-modal approaches that encompass the above “deterrent” factors. Provision of multi-sensory stimulations to offset these negative factors are varied and have focused on tactile, auditory, and olfactory senses, e.g., skin-to-skin holding/kangaroo care, infant massage/tactile-kinesthetic, music therapy, maternal pheromones. Except for the benefits of the well-acknowledged skin-to-skin holding (82, 83), music and massage therapy along with maternal pheromones will require further confirmation (84–86). We examined the potential benefits of some of these interventions on infant oral feeding difficulties as few of them have been used for this purpose. The OFS scale was used as an index of oral feeding skills, while days from initiation to independent oral feeding was used as index of oral feeding performance. We demonstrated the benefits of a specific *non-nutritive oral motor training* protocol *(NNOMT)* and a *swallow exercis*e directed at sucking and swallowing, respectively (10, 87–89). The use of a protocol consisting of active non-nutritive sucking on a pacifier that followed the same schedule as that of the NNOMT did not replicate the benefit observed with the latter (89). As the general benefits of infant massage for infants and parents are well-recognized (90, 91), we verified its beneficial effect on preterm infants' oral feeding using the *massage protocol* developed by Field for preterm infants (92). This intervention was similarly effective on improving infants' OFS levels and accelerating their attainment of independent oral feeding (87). Over the years, various infant feeding positions have been advocated as “optimal,” e.g., the customary semi-reclined (control), upright, and sidelying. As each lacks evidence-based support, we examined VLBW infants' performance when fed under these three conditions. No statistical difference in transition time from tube to independent oral feeding was observed (28). It should be noted that no infants in the above studies demonstrated any adverse events during their feedings.

From a common sense approach, as poor endurance is commonly linked to poor feeding performance, “*endurance training*” is suggested whereby a feeding duration is stopped when the infant shows signs of fatigue, e.g., increased pauses, changes in organization or state, rather than “encouraging” the infant to continue. For instance, if an infant is following a regimen of 2 oral feedings/day (20 min/feeding), but cannot continue after 10 min, a revised schedule of 4 oral feedings/day (10 min/feeding) regimen may be more appropriate and advantageous. Indeed, one may reason that the duration of “practice” time is the same, i.e., 40 min/day, but training during these 40 min in the latter case occurs at a time when the infant has greatest endurance. This principle addresses the common saying that “practice makes perfect” *as long as* the practice duration remains productive. This premise is based on the appreciation that brain plasticity can reorganize sensori-motor areas following beneficial and detrimental practices (93, 94). Consequently, to optimize beneficial sensory inputs, if fatigue were recognized and oral feeding regimen were revised accordingly, detrimental consequences such as oral feeding aversion, regurgitation, aspiration/penetration, may be reduced. Along this line of reasoning, it is proposed that the ISP bottle may be considered an appropriate intervention as it gives control of the feeding to the infants. With control of flow rate and pauses, infants would experience optimal sensory inputs.

## What Can We Do?

### Flexible *Individualized* Management Guidelines

Customarily, the evidence-based efficacy of any intervention on a particular population is confirmed by following appropriately designed studies or randomized clinical trials using appropriate statistical approaches. However, as the functional maturation profiles of preterm infants of same GA and PMA are not uniform, as shown in Figure 3C, one ought not presume that if an intervention does not demonstrate statistical efficacy for a group of infants, that it cannot be efficacious for some of them. Insofar as the care of healthcare providers is directed toward *individual* patients, it is advanced that the ease-of-use of the OFS scale can help identify *individual* infants who do or do not benefit from a particular intervention. A fitting example relates to our earlier study on “optimal” feeding positions wherein no statistical difference in transition time from tube to independent oral feeding was observed between positions (28). If the OFS scale had been used to follow *individual* infants' progress as they transition from tube to independent oral feeding, identification of the ones who did and did not benefit from being fed semi-reclined, upright, or sidelying could have been identified. As such, one of the advantages of the OFS scale is to allow close monitoring of the efficacy of any intervention on *individual* infants (10, 89). It is based on the simplicity of use of the OFS scale that the *individualized* management protocol below is presented. This approach is similar to the one used by feeding therapists in the development of their management plans with the advantage that monitoring performance with the OFS scale will provide objective outcome measures that have been shown to correlate with proficiency, endurance, oral feeding skills, and performance (Figures 3A,B).

**Figure 5:**
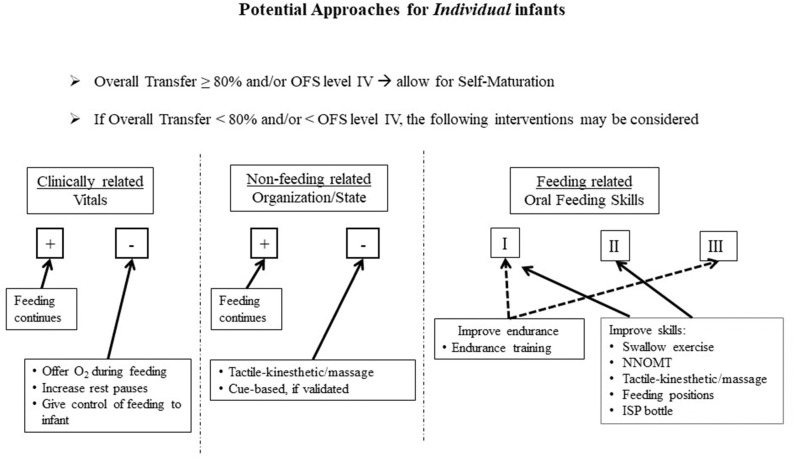
Proposed approaches based on *individual* infant's challenges, i.e., Clinically related, Non-feeding related, Feeding related; (+) Adequate, (–) Inadequate; OFS levels I, II, III.

**Figure 6:**
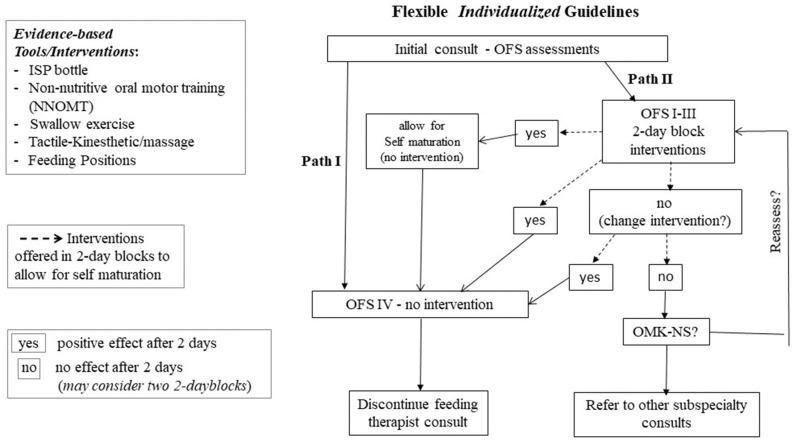
Flexible *individualized* guidelines based on OFS levels; Path I for OFS level IV and Overall Transfer > 80%; Path II for OFS level I to III and Overall Transfer <80%; interventions provided in 2-day blocks; (yes) positive effect; (no) no effect after 2-day block (see text for details).

Consequently, it is advanced that the routine use of the OFS scale will allow caregivers to closely monitor the oral feeding skills of their *individual* patients. The guidelines described below make use of a “research-to-practice” translation to complement the current clinical practices. Although we do not know the initial causes of *individual* infants' dysphagia, we do know that they may arise from their clinical status, non-feeding, and feeding related factors, e.g., O_2_ desaturation, behavioral organization/state, immature oral feeding skills, respectively. For each of these three categories, there are evidence-based beneficial interventions available as presented earlier (Figure 5). Proficiency can be used as an estimate of infants' nutritive sucking ability when fatigue is minimal, while rate of milk transfer can be used as an estimate of infants' endurance *as a function* of their clinical status, behavioral organization/state, and/or stress (95–99). Thus, considering all the factors that may impact preterm infants' ability in attaining independent oral feeding, Figures 5, 6 are proposed targeted interventions and general guidelines that may be considered in the management plans of *individual* NICU infants. As the quality of their feeding performance is primarily measured clinically by their % overall transfer, Figure 5 proposes that with overall transfer ≥ 80% or OFS level IV, no interventions are proposed to allow for infant “self-maturation.” With overall transfer <80% and/or OFS I–III, interventions may be offered to support infants' feeding performance based on their clinically related vitals, non-feeding related organization/state, and/or feeding skills as assessed by their OFS levels. Under each of these categories, targeted interventions are proposed based on clinical practices and objective evidence-based studies presented earlier. In regard to feeding related factors, proposed interventions are based on the interpretation of the OFS levels presented in Figure 3B.

Figure 6 is a suggested *flexible* algorithm using the OFS scale as the “monitoring device.” The protocol calls for “reassessments” in 2-day block of times to allow for infants “self-maturation.” This 2-day block window is based on our clinical observations that some infants, on their own, can show improvement in their oral feeding aptitude within 2 days in the absence of any interventions. Two paths are described. At a feeding therapist's initial bottle feeding consult, if infants demonstrate an OFS level IV and/or overall transfer ≥ 80% (Path I), progression of oral feeding would be based on the provider/team's recommendation in oral feeding advancement with no needed intervention. With OFS levels I to III or overall transfer <80% (Path II), infants will be offered targeted interventions listed in Figure 5 based upon their clinical stability, organization/state, and/or OFS skill levels. It should be reminded that monitoring infants' OFS levels requires minimal effort as proficiency can be computed at any feeding session by simply measuring the volume taken during the first 5 min at a “burping” pause in addition to the overall transfer and feeding duration that are customarily collected by caregivers. Daily intervention frequency and duration can be offered based on *individual* infants' clinical status, e.g., 15 min/day and reassessed in 2-day blocks. If a positive effect is observed in either OFS levels or % overall transfer, infant can be moved into path I *when* OFS level IV or overall performance ≥ 80% is achieved. If no progress is observed after two 2-day blocks, i.e., 4 days of intervention, a change in intervention may be appropriate as the earlier intervention may not have targeted the appropriate level of the Swallow Process. An assessment using the OMK-NS monitoring system may be recommended in order to directly assess the maturation stage of infants' nutritive skills and suck-swallow-respiratory coordination if necessary. Referral to other pediatric subspecialties, e.g., Pediatric Ear Nose and Throat, Gastroenterology, Pulmonology, Cardiology, may be recommended. Any regression in oral feeding performance would naturally require further medical examination as it may be due to “yet” undetected clinical issues, e.g., sepsis, GI issues, silent gastro-esophageal reflex. The “flexibility” of the proposed guidelines is based on the recognition that due to the broad variations in preterm infants' developmental profiles, caregivers ought to remain flexible in the management plans they devise.

As mentioned above, the focus placed on bottle feeding described in our work does not reflect any partiality for bottle- over breast-feeding, but rather a first step at understanding the development of infants' own skills in the absence of any potential maternal input(s) that may occur during breastfeeding, e.g., poor lactation, maternal own comfort to breastfeeding, infant proper attachment to the nipple-areolar complex. The OFS assessments in the NICU does not threaten infants' breastfeeding opportunities as most infants who are primarily breastfeeding are bottle fed when mother is not present. In fact, their OFS levels may help parents and caregivers identify potential breastfeeding difficulties resulting from infant's proficiency and endurance (Figure 3A). One may speculate that improvement of these infants' OFS levels may also lead to improved breastfeeding skills, e.g., latching-on, and earlier breastfeeding success.

Insofar as our tools and intervention programs can be readily used by healthcare providers in hospitals as well as out-patient settings, it is advanced that their adoption in clinics could provide a “Continuum of Care” approach that would allow “feeders and growers” to be discharged earlier from NICU and reduce medical costs, as such care can be readily assumed by out-patient services offering the same oral feeding management protocol.

In brief, the intent of these proposed flexible guidelines is to introduce to clinicians the evidence-based beneficial tools and interventions developed through research over recent years that could benefit the care of their high-risk patients with oral feeding challenges.

### Caveats

It is recognized that the knowledge we gained from our research will require confirmation via additional well-designed studies. However, the above proposed guidelines are based on the following justifications:

1. Preterm infants' difficulties in attaining safe and efficient oral feeding lead to prolonged NICU hospitalization, increased maternal stress, and medical costs. As such, the sooner they attain independent oral feeding safely and efficiently, the better.
2. Due to our limited understanding of the cause(s) leading to oral feeding difficulties, the current practices provided by feeding therapists lack evidence-based backing and consensus from team members as any benefit may be due to the infant's normal maturation.
3. In general, any new treatment/therapy requires additional well-designed studies to confirm and validate their efficacy. Insofar as our proposed guidelines address the care of *individual* infants rather than groups of infants, it is advanced that such approach may not be relevant. As mention earlier, we do not yet have the technology to determine the origination site(s) of an oral feeding problem in the Swallow Process and thus cannot identify the appropriate targeted intervention(s) to best treat them. For instance, if the adverse events observed on a particular patient originated at the “Safety” level, i.e., Respiration-Pharyngeal phase of swallowing (Figure 1), providing NNOMT treatment would likely not show any benefit. As such, providers need to remember that if a particular intervention is not efficacious for one infant, it can be for another as the cause(s) of their dysphagia may lie at different levels of the Swallow Process. This is the reason why in the proposed algorithm (Figure 6), a “trial and error approach” is used as different targeted interventions would be offered in two 2-day blocks, if no benefits are observed.
4. We speculate that the development of *individualized* management care plans that combine current feeding therapists' practices with the tools and interventions presented would lead to greater success for infants and the team members caring for them.

## Conclusion

In summary, this report presents a review of the practices currently offered to NICU infants facing oral feeding challenges and the latest research that led to a number of efficacious evidence-based tools and interventions. It is recognized that “the underlying principles of the above model [will need further validation in order] to be clearly disseminated to practitioners of this field” (Research Model Innovations in Advancing Neonatal Care). However, it is hoped that in providing a greater understanding of the potential causes at the root of preterm infants' oral feeding difficulties, our research will improve the current clinical practice and assist “in the development of [additional] diagnostic tools and new therapies.” This review presents a new perspective of how combining features of current practices with the use of novel tools/interventions could help practitioners improve their patients' care in developing structured and innovative management plans catered to the *specific* needs of *individual* patient. Additionally, it is proposed that a “Continuum of Care” approach may be envisaged whereby “feeders and growers” could be followed by out-patient clinical or home care services using the same technologies and methodologies. This would allow for earlier discharge, family reunion, and reduced medical cost for all.

## Data Availability Statement

The raw data supporting the conclusions of this article will be made available by the authors, without undue reservation, to any qualified researcher.

## Ethics Statement

The studies involving human participants were reviewed and approved by The Baylor College of Medicine Institutional Review Board for Human Research. Parental consent was obtained following approval by attending neonatologists. Written informed consent to participate in this study was provided by the participants' legal guardian/next of kin.

## Author Contributions

CL is the primary investigator of the above series of studies and was involved in all aspects of the research, i.e., study designs, team members supervision, data collection, statistical analyses, and interpretations, preparation of manuscripts.

## Conflict of Interest

The author declares that the research was conducted in the absence of any commercial or financial relationships that could be construed as a potential conflict of interest.
